# Supplementary material for: Heparin-based hydrogel scaffolding alters the transcriptomic profile and increases the chemoresistance of MDA-MB-231 triple-negative breast cancer cells
Source: Biomater Sci. 2020 Feb 13;8(10):2786–96. doi: 10.1039/c9bm01481k (PMC7497406; doi:10.1039/c9bm01481k)
Supplement: Supplementary file 2 [file BM-008-C9BM01481K-s002.zip › Supplementary File 4/HP-BhydrogelvControl/Pathways/my_analysis.Gsea.1545201079907/HALLMARK_XENOBIOTIC_METABOLISM.html]

Details for gene set HALLMARK\_XENOBIOTIC\_METABOLISM[GSEA]

|  || Dataset | expr.class.cls#HYD\_versus\_CONTROL.class.cls#HYD\_versus\_CONTROL\_repos |
| Phenotype | class.cls#HYD\_versus\_CONTROL\_repos |
| Upregulated in class | CONTROL |
| GeneSet | HALLMARK\_XENOBIOTIC\_METABOLISM |
| Enrichment Score (ES) | -0.25194257 |
| Normalized Enrichment Score (NES) | -1.1697501 |
| Nominal p-value | 0.115 |
| FDR q-value | 0.17332597 |
| FWER p-Value | 0.83 |
Table: GSEA Results Summary

  

Fig 1: Enrichment plot: HALLMARK\_XENOBIOTIC\_METABOLISM      
 Profile of the Running ES Score & Positions of GeneSet Members on the Rank Ordered List

  

| PROBE | DESCRIPTION (from dataset) | GENE SYMBOL | GENE\_TITLE | RANK IN GENE LIST | RANK METRIC SCORE | RUNNING ES | CORE ENRICHMENT || 1 | CSAD | na |  |  | 93 | 3.328 | 0.0247 | No |
| 2 | CYP2E1 | na |  |  | 221 | 2.868 | 0.0436 | No |
| 3 | GCKR | na |  |  | 513 | 2.326 | 0.0490 | No |
| 4 | LCAT | na |  |  | 536 | 2.295 | 0.0683 | No |
| 5 | SLC12A4 | na |  |  | 621 | 2.183 | 0.0833 | No |
| 6 | LONP1 | na |  |  | 988 | 1.881 | 0.0808 | No |
| 7 | UPP1 | na |  |  | 1307 | 1.685 | 0.0791 | No |
| 8 | ID2 | na |  |  | 1383 | 1.637 | 0.0898 | No |
| 9 | BCAR1 | na |  |  | 1577 | 1.541 | 0.0934 | No |
| 10 | ATOH8 | na |  |  | 1626 | 1.521 | 0.1044 | No |
| 11 | PPARD | na |  |  | 2220 | 1.265 | 0.0845 | No |
| 12 | ASL | na |  |  | 2248 | 1.258 | 0.0943 | No |
| 13 | EPHA2 | na |  |  | 2560 | 1.156 | 0.0882 | No |
| 14 | PINK1 | na |  |  | 2820 | 1.080 | 0.0843 | No |
| 15 | ITIH4 | na |  |  | 2982 | 1.042 | 0.0851 | No |
| 16 | HGFAC | na |  |  | 3113 | 1.006 | 0.0872 | No |
| 17 | ACOX3 | na |  |  | 3597 | 0.894 | 0.0698 | No |
| 18 | HES6 | na |  |  | 3700 | 0.870 | 0.0722 | No |
| 19 | FAH | na |  |  | 3746 | 0.859 | 0.0775 | No |
| 20 | HACL1 | na |  |  | 3848 | 0.842 | 0.0797 | No |
| 21 | GSS | na |  |  | 3950 | 0.822 | 0.0817 | No |
| 22 | ABCC3 | na |  |  | 4004 | 0.811 | 0.0861 | No |
| 23 | SLC1A5 | na |  |  | 4248 | 0.762 | 0.0802 | No |
| 24 | DHPS | na |  |  | 4272 | 0.757 | 0.0857 | No |
| 25 | CYP1A1 | na |  |  | 4586 | 0.695 | 0.0754 | No |
| 26 | DHRS7 | na |  |  | 4798 | 0.659 | 0.0702 | No |
| 27 | XDH | na |  |  | 4840 | 0.651 | 0.0739 | No |
| 28 | MPP2 | na |  |  | 4914 | 0.640 | 0.0757 | No |
| 29 | ACP2 | na |  |  | 5041 | 0.620 | 0.0746 | No |
| 30 | PMM1 | na |  |  | 5293 | 0.586 | 0.0667 | No |
| 31 | POR | na |  |  | 5382 | 0.572 | 0.0671 | No |
| 32 | VTN | na |  |  | 5473 | 0.557 | 0.0674 | No |
| 33 | GNMT | na |  |  | 5481 | 0.555 | 0.0719 | No |
| 34 | FBLN1 | na |  |  | 5556 | 0.544 | 0.0729 | No |
| 35 | CYFIP2 | na |  |  | 5787 | 0.515 | 0.0654 | No |
| 36 | GAD1 | na |  |  | 5904 | 0.499 | 0.0638 | No |
| 37 | GCH1 | na |  |  | 6157 | 0.463 | 0.0546 | No |
| 38 | ETS2 | na |  |  | 6164 | 0.462 | 0.0584 | No |
| 39 | ATP2A2 | na |  |  | 6241 | 0.453 | 0.0585 | No |
| 40 | RAP1GAP | na |  |  | 6262 | 0.449 | 0.0614 | No |
| 41 | MAN1A1 | na |  |  | 6499 | 0.416 | 0.0527 | No |
| 42 | SMOX | na |  |  | 6502 | 0.415 | 0.0563 | No |
| 43 | DCXR | na |  |  | 6794 | 0.384 | 0.0445 | No |
| 44 | LEAP2 | na |  |  | 7349 | 0.320 | 0.0182 | No |
| 45 | AP4B1 | na |  |  | 7571 | 0.297 | 0.0093 | No |
| 46 | NMT1 | na |  |  | 7640 | 0.286 | 0.0083 | No |
| 47 | CYP2S1 | na |  |  | 7751 | 0.281 | 0.0050 | No |
| 48 | APOE | na |  |  | 8142 | 0.241 | -0.0134 | No |
| 49 | ABCC2 | na |  |  | 8198 | 0.233 | -0.0142 | No |
| 50 | GSTT2 | na |  |  | 8215 | 0.229 | -0.0130 | No |
| 51 | SLC6A6 | na |  |  | 8243 | 0.227 | -0.0124 | No |
| 52 | PSMB10 | na |  |  | 8309 | 0.219 | -0.0138 | No |
| 53 | CROT | na |  |  | 8403 | 0.207 | -0.0169 | No |
| 54 | ALDH3A1 | na |  |  | 8629 | 0.188 | -0.0270 | No |
| 55 | COMT | na |  |  | 8845 | 0.160 | -0.0369 | No |
| 56 | AKR1C2 | na |  |  | 8870 | 0.156 | -0.0367 | No |
| 57 | CNDP2 | na |  |  | 8995 | 0.155 | -0.0419 | No |
| 58 | GCLC | na |  |  | 9105 | 0.140 | -0.0463 | No |
| 59 | KYNU | na |  |  | 9128 | 0.137 | -0.0463 | No |
| 60 | DDT | na |  |  | 9271 | 0.121 | -0.0527 | No |
| 61 | PYCR1 | na |  |  | 9476 | 0.093 | -0.0625 | No |
| 62 | ECH1 | na |  |  | 9762 | 0.069 | -0.0769 | No |
| 63 | EPHX1 | na |  |  | 10016 | 0.038 | -0.0898 | No |
| 64 | CYP27A1 | na |  |  | 10067 | 0.034 | -0.0922 | No |
| 65 | CFB | na |  |  | 10199 | 0.015 | -0.0989 | No |
| 66 | GCNT2 | na |  |  | 10333 | 0.014 | -0.1057 | No |
| 67 | RETSAT | na |  |  | 10420 | 0.005 | -0.1102 | No |
| 68 | SERTAD1 | na |  |  | 10449 | 0.001 | -0.1117 | No |
| 69 | NINJ1 | na |  |  | 10608 | -0.017 | -0.1198 | No |
| 70 | AOX1 | na |  |  | 10647 | -0.023 | -0.1216 | No |
| 71 | VNN1 | na |  |  | 10879 | -0.050 | -0.1333 | No |
| 72 | JUP | na |  |  | 11065 | -0.073 | -0.1424 | No |
| 73 | IGFBP1 | na |  |  | 11345 | -0.095 | -0.1562 | No |
| 74 | TNFRSF1A | na |  |  | 11403 | -0.101 | -0.1582 | No |
| 75 | MTHFD1 | na |  |  | 11427 | -0.104 | -0.1585 | No |
| 76 | PTGES | na |  |  | 11658 | -0.133 | -0.1694 | No |
| 77 | PC | na |  |  | 12058 | -0.180 | -0.1888 | No |
| 78 | KARS | na |  |  | 12158 | -0.193 | -0.1922 | No |
| 79 | SLC35B1 | na |  |  | 12210 | -0.200 | -0.1931 | No |
| 80 | CYP2J2 | na |  |  | 12481 | -0.222 | -0.2053 | No |
| 81 | AHCY | na |  |  | 12587 | -0.232 | -0.2088 | No |
| 82 | ACO2 | na |  |  | 12768 | -0.255 | -0.2160 | No |
| 83 | FAS | na |  |  | 12828 | -0.261 | -0.2167 | No |
| 84 | SSR3 | na |  |  | 12910 | -0.273 | -0.2186 | No |
| 85 | IL1R1 | na |  |  | 13023 | -0.288 | -0.2219 | No |
| 86 | GSTM4 | na |  |  | 13354 | -0.330 | -0.2363 | No |
| 87 | FBP1 | na |  |  | 13369 | -0.333 | -0.2340 | No |
| 88 | NFS1 | na |  |  | 13456 | -0.346 | -0.2355 | No |
| 89 | SHMT2 | na |  |  | 13483 | -0.351 | -0.2337 | No |
| 90 | PEMT | na |  |  | 13524 | -0.357 | -0.2326 | No |
| 91 | TTPA | na |  |  | 13561 | -0.362 | -0.2313 | No |
| 92 | ADH5 | na |  |  | 13562 | -0.363 | -0.2281 | No |
| 93 | DDAH2 | na |  |  | 13726 | -0.392 | -0.2332 | No |
| 94 | SLC46A3 | na |  |  | 13732 | -0.393 | -0.2299 | No |
| 95 | NPC1 | na |  |  | 13743 | -0.394 | -0.2269 | No |
| 96 | BPHL | na |  |  | 13805 | -0.404 | -0.2265 | No |
| 97 | UPB1 | na |  |  | 13850 | -0.411 | -0.2252 | No |
| 98 | PLG | na |  |  | 14094 | -0.429 | -0.2341 | No |
| 99 | SLC35D1 | na |  |  | 14339 | -0.469 | -0.2428 | No |
| 100 | TPST1 | na |  |  | 14443 | -0.482 | -0.2439 | No |
| 101 | TGFB2 | na |  |  | 14509 | -0.492 | -0.2429 | No |
| 102 | ACOX1 | na |  |  | 14591 | -0.504 | -0.2427 | No |
| 103 | MARCH6 | na |  |  | 14599 | -0.506 | -0.2386 | No |
| 104 | FMO3 | na |  |  | 14855 | -0.544 | -0.2471 | Yes |
| 105 | PGRMC1 | na |  |  | 14887 | -0.547 | -0.2439 | Yes |
| 106 | NQO1 | na |  |  | 14968 | -0.557 | -0.2431 | Yes |
| 107 | PTGES3 | na |  |  | 14997 | -0.561 | -0.2396 | Yes |
| 108 | BCAT1 | na |  |  | 15072 | -0.574 | -0.2384 | Yes |
| 109 | GART | na |  |  | 15215 | -0.599 | -0.2405 | Yes |
| 110 | PDK4 | na |  |  | 15248 | -0.606 | -0.2368 | Yes |
| 111 | HMOX1 | na |  |  | 15431 | -0.638 | -0.2406 | Yes |
| 112 | CDA | na |  |  | 15484 | -0.647 | -0.2376 | Yes |
| 113 | PTS | na |  |  | 15494 | -0.649 | -0.2323 | Yes |
| 114 | ACOX2 | na |  |  | 15508 | -0.652 | -0.2272 | Yes |
| 115 | GSR | na |  |  | 15538 | -0.659 | -0.2228 | Yes |
| 116 | BLVRB | na |  |  | 15546 | -0.661 | -0.2173 | Yes |
| 117 | ENTPD5 | na |  |  | 15629 | -0.674 | -0.2156 | Yes |
| 118 | ABHD6 | na |  |  | 15722 | -0.686 | -0.2144 | Yes |
| 119 | GABARAPL1 | na |  |  | 15826 | -0.709 | -0.2135 | Yes |
| 120 | DHRS1 | na |  |  | 15944 | -0.732 | -0.2131 | Yes |
| 121 | PAPSS2 | na |  |  | 15945 | -0.732 | -0.2066 | Yes |
| 122 | ALDH2 | na |  |  | 15979 | -0.740 | -0.2017 | Yes |
| 123 | CBR1 | na |  |  | 16064 | -0.762 | -0.1994 | Yes |
| 124 | SPINT2 | na |  |  | 16253 | -0.799 | -0.2021 | Yes |
| 125 | ALAS1 | na |  |  | 16364 | -0.825 | -0.2006 | Yes |
| 126 | LPIN2 | na |  |  | 16674 | -0.892 | -0.2088 | Yes |
| 127 | ARG2 | na |  |  | 16728 | -0.907 | -0.2035 | Yes |
| 128 | CYB5A | na |  |  | 16738 | -0.910 | -0.1959 | Yes |
| 129 | TMBIM6 | na |  |  | 16758 | -0.916 | -0.1888 | Yes |
| 130 | PTGR1 | na |  |  | 17060 | -1.004 | -0.1956 | Yes |
| 131 | MT2A | na |  |  | 17171 | -1.038 | -0.1922 | Yes |
| 132 | PGD | na |  |  | 17287 | -1.079 | -0.1886 | Yes |
| 133 | PDLIM5 | na |  |  | 17595 | -1.194 | -0.1941 | Yes |
| 134 | TMEM97 | na |  |  | 17777 | -1.277 | -0.1922 | Yes |
| 135 | MCCC2 | na |  |  | 17802 | -1.287 | -0.1820 | Yes |
| 136 | SAR1B | na |  |  | 17921 | -1.341 | -0.1763 | Yes |
| 137 | ETFDH | na |  |  | 17960 | -1.356 | -0.1662 | Yes |
| 138 | ALDH9A1 | na |  |  | 18095 | -1.437 | -0.1605 | Yes |
| 139 | IDH1 | na |  |  | 18161 | -1.475 | -0.1508 | Yes |
| 140 | MAOA | na |  |  | 18217 | -1.509 | -0.1402 | Yes |
| 141 | AKR1C3 | na |  |  | 18337 | -1.592 | -0.1323 | Yes |
| 142 | UGDH | na |  |  | 18399 | -1.641 | -0.1209 | Yes |
| 143 | CASP6 | na |  |  | 18493 | -1.701 | -0.1106 | Yes |
| 144 | ELOVL5 | na |  |  | 18504 | -1.707 | -0.0960 | Yes |
| 145 | ACP1 | na |  |  | 18505 | -1.708 | -0.0808 | Yes |
| 146 | HSD11B1 | na |  |  | 18701 | -1.893 | -0.0741 | Yes |
| 147 | PROS1 | na |  |  | 18766 | -1.970 | -0.0600 | Yes |
| 148 | HPRT1 | na |  |  | 18898 | -2.164 | -0.0476 | Yes |
| 149 | GSTO1 | na |  |  | 18916 | -2.201 | -0.0289 | Yes |
| 150 | ARPP19 | na |  |  | 19008 | -2.369 | -0.0126 | Yes |
| 151 | CAT | na |  |  | 19052 | -2.529 | 0.0077 | Yes |
Table: GSEA details [plain text format]

  

Fig 2: HALLMARK\_XENOBIOTIC\_METABOLISM      
 Blue-Pink O' Gram in the Space of the Analyzed GeneSet

  

Fig 3: HALLMARK\_XENOBIOTIC\_METABOLISM: Random ES distribution      
 Gene set null distribution of ES for **HALLMARK\_XENOBIOTIC\_METABOLISM**
